# Native and alien species suffer from late arrival, while negative effects of multiple alien species on natives vary

**DOI:** 10.1007/s00442-021-05017-3

**Published:** 2021-08-19

**Authors:** Viktoria Ferenc, Christian Merkert, Frederik Zilles, Christine S. Sheppard

**Affiliations:** Institute of Landscape and Plant Ecology, University of Hohenheim, 70593 Stuttgart, Germany

**Keywords:** Pot experiment, Competition, Invasional meltdown, Native-alien interaction, Priority effect

## Abstract

Ongoing globalisation and climate change are causing plant species to invade new habitats and thereby alter biodiversity and ecosystem functioning. Since numbers of plant invasions continue to increase globally, it is crucial to investigate the effects of multiple co-occurring alien species on native communities. Furthermore, priority effects due to the earlier emergence of certain species affecting fitness of later arriving species can shape community structure and affect native species performance. We investigate in a common garden pot experiment the interactions among five alien-native species pairs. First we focus on the effect of growing with either one or two alien neighbour species on a native plant, second we alter the arrival time of the alien or native neighbour by 3 weeks. Generally, native species performance decreased when surrounded by two alien species compared to only one, although the magnitude of this effect varied depending on species, with one species even performing better with alien neighbours than in monoculture. Species performance greatly decreased when arriving second in the pot, for both native and alien species. In contrast, alien species tended to benefit more from arriving early. Given that we studied annual ruderal species, their potentially lower competitive ability might explain why we detected negative effects of late arrival. We highlight the need to further elucidate underlying mechanisms of small-scale invasion dynamics to achieve generalisations concerning the response of multiple alien and native plants given their species-specific differences in response to neighbour species and arrival time.

**Supplementary Information:**

The online version contains supplementary material available at 10.1007/s00442-021-05017-3.

## Introduction

Invasive species pose a major threat to biodiversity and ecosystem functioning (Pyšek et al. 2020). Global analyses show an increase in the number of invasive species (Seebens et al. 2017, 2018), indicating that the negative impacts of invasive species already observed might become even more severe in the future. Increasing numbers of alien species also raises attention to the importance of examining interactions of co-occurring alien species and how they may affect natives, which was until recently an often neglected aspect of invasion studies (Kuebbing et al. 2013).

Invasive species are often found to be better competitors than native species (Vila and Weiner 2004) as the traits of invaders tend to lead to higher fitness than natives (Ordonez et al. 2010; van Kleunen et al. 2010). In comparisons to single alien-native pairs, the effect of more than one alien species being present has received far less attention. When multiple aliens are present, the following mechanisms might lead to stronger negative effects on a co-occurring native species: asymmetric competition meaning that the alien species compete less with each other than with the native species (Kuebbing and Nuñez 2016), or even facilitation among alien species (Flory and Bauer 2014) leading to higher negative impacts on the native species. The outcome of such an effect was termed ‘invasional meltdown’ by Simberloff and von Holle (1999).

Besides more favorable trait values related to superior competitive ability explaining alien species advantage over natives, earlier emergence (as commonly observed for alien species) may affect growth, development and reproduction of later arriving native plants, termed priority effects (Young et al. 2001). According to von Gillhaussen et al. (2014), priority effects occur if a species arriving first affects the fitness of a later arriving species. Such effects can strongly influence plant community structure and functioning, even after multiple seasons (Weidlich et al. 2018). The effect can be caused by a direct competitive advantage during the growing period or indirectly, either by reducing resource availability or by residues in the soil such as allelochemicals that interfere with the subsequently arriving species, so called soil legacies (Grman and Suding 2010). Priority effects whereby a later arriving species is affected by the earlier arriving one can not only occur if one species arrives later in a certain site than the other species, but also if all seeds are present already but species germinate at different times (Wainwright et al. 2012). Alien species tend to germinate earlier and more successfully than native species (Chrobock et al. 2011; Wainwright and Cleland 2013) as well as starting to grow earlier (Wilsey et al. 2011). Consequently, a prior establishment of alien species has been found to negatively impact native species (Dickson et al. 2012; Delory et al. 2019a). In contrast, it is possible that late-arriving alien species may actually suffer less from the earlier arrival of natives (Stuble and Souza 2016), as alien species tend to have higher competitive ability and growth rates (Levine et al. 2003; van Kleunen et al. 2010). Furthermore, arriving late may even result in a positive priority effect, depending on whether the species is alien or native and to which functional group it belongs (Delory et al. 2019b). For instance, legume species fix nitrogen and enrich soil (Temperton et al. 2007), which may benefit subsequently arriving species.

In this study, we thus aim to find out how these two important aspects of invasion success, the effects of co-occurring aliens and priority effects, affect natives. To this end, we conducted a common garden pot experiment using five alien and five native species of different families. In the first part of the experiment, we investigate the effect of two alien species relative to only one alien species (neighbour experiment) on the growth of five different native species. We expect higher performance of native plants when having one alien neighbour species compared to having two alien neighbour species, as possible synergistic negative effects are not present. In the second part of our experiment, we manipulate the order of arrival of alien and native species to investigate potential priority effects among different alien-native species pairs (priority experiment). We hypothesize that species generally benefit from arriving early but suffer from arriving late (compared to arriving simultaneously). However, we expect the advantage of arriving first compared to simultaneously to be stronger for alien species which often have ruderal strategies and germinate and grow fast. This might either be due to their often stronger competitive ability helping them to compensate for the disadvantage, or because the priority effects of the native species arriving first are weaker.

## Material and methods

### Study species

Our study included ten annual plant species consisting of five confamilial alien-native species pairs (Table 1). We chose the specific plant families to include three different functional groups (forbs: Amaranthaceae, Asteraceae, and Solanaceae; grasses: Poaceae; and legumes: Fabaceae) to cover a wide range of different species, but also these families are common in central Europe and comprise many alien and invasive species. For the alien-native species pairs, we specifically chose natives that are common. While all five native species are widespread in Germany (52–98% of area occupied in Germany), all alien species are known to be established neophytes (Jäger 2017) but some of them are not (yet) widespread (5–70% of area occupied in Germany, Table 1). As the German floristic literature (Footnote Table 1) is inconsistent in categorising the species in native or archaeophytes, we also considered archaeophytes (*Solanum nigrum, Setaria pumila, Chenopodium album*) as native species, given that their long history in central Europe makes it often difficult to distinguish between these categories (Scholz 2007). All species occur in similar habitats (as ruderal and segetal annuals), and hence they all can principally co-occur. Since such ruderal sites, commonly occurring near human disturbances, are often dominated by alien species and experience high alien seed input (Chytrý et al. 2008), this is an ideal study system to investigate the effect of several co-occurring alien neighbours and effects of shifted arrival times on species performance. The seeds for the experiment were obtained from our own collections of monocultures in previous experiments or from a botanical garden in our region (except for *Vicia villosa* from a commercial supplier, Table 1).

**Table 1:** Status in Germany, seed origin, range size and year of first record for the five study species

Family	Species	Status in Germany	Seed origin	Range size in %	Year of first record
Amaranthaceae	*Amaranthus retroflexus* L	Alien	Previous experiment	69.5	1815
	*Chenopodium album* L	Native	Botanical garden University of Konstanz	92.8	
Asteraceae	*Centaurea diffusa* Lam	Alien	Previous experiment	4.8	1876
	*Lapsana communis* L	Native	Previous experiment	98.0	
Fabaceae	*Vicia villosa* Roth s. l	Alien	Revierberatung Wolmersdorf GmbH & Co. KG, Nindorf, Germany	66.4	1808
	*Trifolium campestre* Schreb	Native	Botanical garden University of Konstanz	92.7	
Poaceae	*Panicum capillare* L	Alien	Previous experiment	13.1	1890
	*Setaria pumila* (Poir.) Roem & Schult	Native	Botanical garden University of Konstanz	52.3	
Solanaceae	*Nicandra physalodes* (L.) J. Gaertn	Alien	Previous experiment	17.2	1782
	*Solanum nigrum* L	Native	Previous experiment	84.5	

Range size was measured as the percentage of occupied area in grid cells at the scale of 11 × 11 km obtained from the database FlorKart (https://doi.org/10.15468/wnkii7) from BfN and NetPhyD Netzwerk Phytodiversität Deutschlands e.V., deutschlandflora.de and ww.floraweb.de). Year of first record and status were obtained from Jäger (2017); www.floraweb.de

### Experimental design

We carried out a common garden experiment during the growing season in 2019 at a field station of the University of Hohenheim (48°42′45.2″ N, 9°11′23.6″ E) in Stuttgart, Germany (400 m a.s.l; mean annual temperature 2019: 10.6 °C; total annual precipitation 2019: 856.5 mm). The experiment consisted of two parts, one assessing the effect of having one or two alien neighbour species on a native target species (“neighbour experiment”) and the other investigating the effect of arriving earlier or later than an alien or native neighbour species (“priority experiment”). For the experiment, we established a total of 360 pots in a completely randomized design. The pots were filled with 10 L of field soil (total soil carbon content 1.77% [0.66% inorganic, 1.11% organic]; total soil nitrogen content 0.059%; 85.5 kg NO3-N/ha and 0.45 kg NH4-N/ha; 205.4 mg P2O5/kg; soil texture of 9.7% clay, 71.9% sand, 18.4% silt) and equipped with a drip watering system.

For the 2 parts of the experiment, seeds of either 1, 2, or 3 of the study species were sown per pot, always using a total of 20 seeds per pot, with 3 replicates for each species or species combination (see below). We established simultaneously sown combinations of an alien and a native species (25 possible combinations with 3 replications resulting in 75 pots with 10 seeds per species), serving as “one neighbour”-treatment for the neighbour experiment and as “simultaneous”-treatment for the priority experiment. To answer the first question on the effect of number of alien neighbour species on a native species, we added a treatment with 2 alien neighbour species, using all 35 possible species combinations with differing alien neighbour functional groups (i.e., forb-grass, forb-legume, legume-grass, with three replicates totalling 105 pots, using 5 seeds of each alien species and 10 seeds of the native species, all sown simultaneously). Additionally, we used monoculture pots of each native target species (sown with 20 seeds, totalling 15 pots) as an intraspecific competition control treatment. To answer the second question on the priority effects of alien and native species, we used all 25 pairwise combinations from the “one neighbour” treatment, whereas we used 2 additional treatments, with each of the species either arriving first or second (totalling 150 pots). For this, we sowed the alien (or the native) species 3 weeks later than the native (or the alien). We sowed seeds of the neighbour experiment as well as the first arrivals of the priority experiment on 22 May 2019 and harvested all plants on 14 Sep 2019 (16 weeks later). Weeds emerging from the seedbank were removed before sowing and regularly during the experimental period.

### Data collection

We counted germinated seedlings 6 weeks after first sowing (3 weeks after second sowing in the priority experiment, respectively) on 26 June 2019. To obtain a more natural population-level experiment and include all life-stages of the individuals we did not replace non-germinated seeds. To measure plant performance, we considered aboveground biomass as proxy for competitive ability and number of flowerheads as a measure of reproductive output and proxy for fitness for annual species (Gaudet and Keddy 1988; Jelbert et al. 2015). Further we observed flowering onset, as phenology is presumably acting independently of general resource strategies (Craine et al. 2012). We recorded flowering of each species per pot weekly throughout the experimental period. Before harvest, we counted the number of individuals as measure for final establishment and the total number of flower heads per pot. We then cut all plants at ground level and dried the aboveground biomass of each species per pot at 70 °C for 72 h.

### Statistical analysis

All statistical analyses were performed using the software R (version 4.0.4, R Core Team 2021). To assess the effect of neighbour treatments on target plants, for biomass and number of flowerheads the relative performance of individuals in interspecific competition (i.e., pots containing two or three species) was calculated as the log response ratio (lnRR, log(performance in mixture/performance in monoculture)) (Weigelt and Jolliffe 2003) which represents the ability to tolerate the respective interspecific treatment compared to growing in monoculture (intraspecific competition). For a better estimate of species overall performance, we used pot-level performance values (i.e., total aboveground biomass per species per pot and total number of flowerheads per species per pot) and divided the performance values in monoculture by two to correct for the double amount of seeds added in monoculture pots per species compared to interspecific treatments. Positive values of the lnRR represent better performance in the respective interspecific treatment while negative values represent better performance in monoculture. In the priority-experiment, we used the (log transformed) absolute performance measures to compare differences in biomass and number of flowerheads depending on arrival time. We further considered flowering onset and establishment success (after 6 weeks and at harvest) as absolute performance measures (only in interspecific treatments) for both parts of the experiment. To ensure that each pot actually represents the species combination it originally was assigned, we included only pots in the analysis where at least one individual of all species that were sown germinated (removal of 85 pots in the neighbour-experiment and 116 pots in the priority experiment for all performance measures except establishment). For the neighbour experiment, we only considered the native species as target, which is growing together with one or two alien species. For the priority experiment, both species in the pot were considered as target species (whereby we analysed the native and alien species separately). We performed a control analysis on average performance per individual in a pot and found no qualitative differences to the population-level (pot) measures which are reported hereafter.

For the neighbour experiment, our main interest was the effect of number of alien neighbour species (one vs two) and differences among target species. We analysed each normally distributed performance measure (lnRR biomass, lnRR flowerheads, flowering onset) with a linear model, while for initial and final establishment we used a generalized linear model with a quasibinomial distribution due to overdispersion. We included the number of alien neighbour species and target species identity as factors, as well as their interaction. To assess the importance of the explanatory factors, we performed backwards step-wise model simplification using F-tests (employing Type III Sum of Squares to account for the unbalanced dataset due to high mortality in some species-combinations) to obtain the minimum adequate model, containing only significant terms (whereby we retained marginally significant effects, i.e., *P* < 0.1). Given that we found a negative effect of having two alien neighbour species (see below), we additionally tested if this effect might be due to more neighbour biomass being produced per pot when aliens grow in a two-species neighbour mixture compared to when growing with a single neighbour. To this end, we calculated the expected biomass in the two-neighbour treatment by adding for the two neighbour species the average of the respective one-neighbour treatment, divided by two (for each neighbour species combination separately). We then performed a one-tailed paired t-test across the seven alien neighbour species combinations comparing the mean biomass production by neighbours in two-neighbour treatment with the expected biomass.

For the priority experiment, our main interest was the order of arrival (first, second, simultaneous) and differences between alien and native targets. We analysed each normally distributed performance measure (log-transformed biomass, log-transformed flowerheads, flowering onset) with a linear mixed effects model within the R package lme4 (Bates et al. 2010), while for initial and final establishment we used a generalized linear model with a quasibinomial distribution due to overdispersion. We included the fixed effects of order of sowing and target species identity as well as their interactions. We used neighbour species identity as a random effect, except for the models for initial and final establishment due to singular fit issues. To assess the importance of fixed effects, we performed backwards step-wise model simplification using likelihood ratio tests (F-tests for establishment, i.e., quasibinomial models) to obtain the minimum adequate model, containing only significant terms.

## Results

### Neighbour experiment

**Fig. 1:**
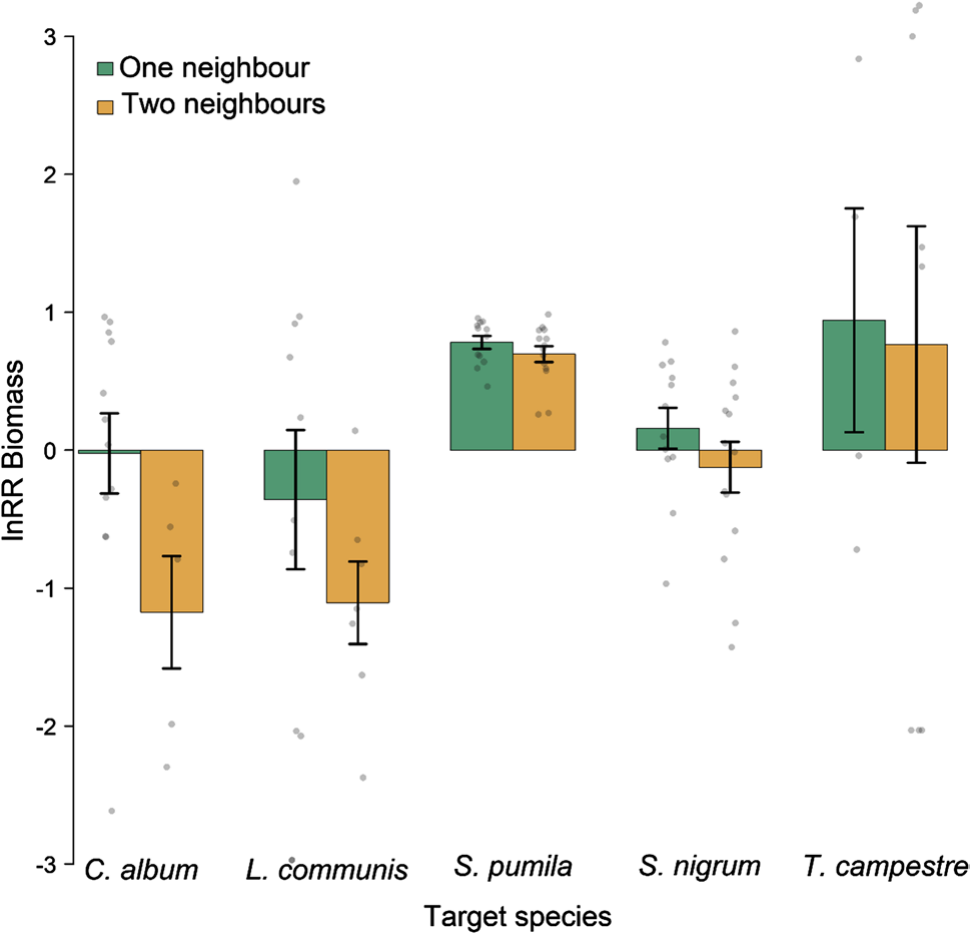
The effect of one vs. two alien neighbor species treatment on the performance of five native target species measured as lnRR biomass. Bars are means ± SE; grey dots depict individual data points. For sample sizes, see Electronic Supplementary Material Table A1

Native target species differed in response to growing with two alien neighbour species compared to only one in terms of lnRR biomass. While over all species the effect of two neighbours compared to one was negative (Table 2), we find *Chenopodium album* and *Lapsana communis* to strongly suffer from having two alien neighbour species (Fig. 1) while *Solanum nigrum* shows a weak negative response. *Setaria pumila* and *Trifolium campestre* showed positive lnRR, indicating better performance in interspecific competition with alien neighbours than in intraspecific competition (although note that for *T. campestre*, performance varied greatly). Despite these apparent species-specific differences, the interaction between species identity and number of alien neighbour species was not significant for lnRR biomass. However, species identity as a main effect was significant for all response measures. To further investigate what caused potential negative effects of having two alien neighbour species on native biomass, we checked whether more neighbour biomass was produced when growing with two neighbours than expected from comparisons with the average biomass of the respective single neighbour treatments. The average biomass produced in the two neighbour treatment was higher than the expected average, which was significant across the seven alien neighbour species combinations (*t* = 4.47; *P* = 0.002; *N* = 7), suggesting a more than additive effect on the target individuals when growing with two compared to one alien neighbour species (Online Resource 1, Fig. A2).

**Table 2:** Results of model selection in the neighbour experiment, showing the significant effects (including marginally significant effects, i.e., *P* < 0.1) retained in the minimum adequate models

Performance measure	Significant effects	Test statistic	*R*^2^_adj_
lnRR biomass	Species No. alien neighbour species	*F*(4, 93) = 7.5; *P* < 0.001 *F*(1, 96) = 3.8; *P* = 0.053	0.21
lnRR flowerheads	Species	F(4, 95) = 11.3; *P* < 0.001	0.30
Initial establishment	Species	F(4, 176) = 67.5; *P* < 0.001	0.60
Final establishment	Species	F(4, 176) = 53.8; *P* < 0.001	0.55
Flowering onset	Species	F(4, 57) = 8.0; *P* < 0.001	0.32

For each model of the five performance measures (lnRR biomass, lnRR flowerheads, initial and final establishment, flowering onset), the significant effects are shown with their respective test statistic and adjusted *R*^2^ of the models. Adjusted *R*^2^ for initial and final establishment was obtained using a Kullback–Leibler-divergence-based method, extended to quasi models by Zhang et al. (2017) using the R package rsq (Zhang 2021)

### Priority experiment

**Fig. 2:**
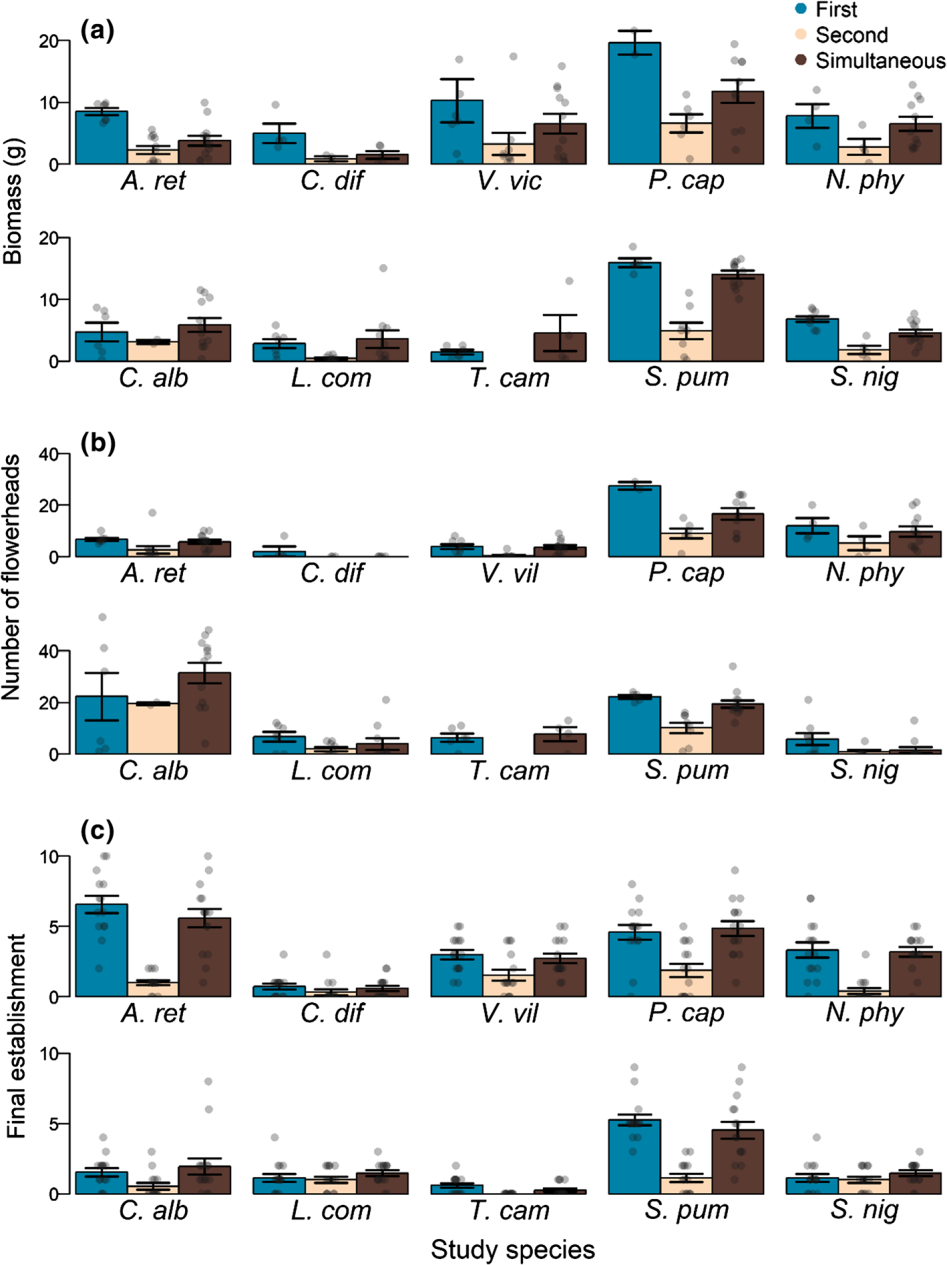
The effects of order of arrival (arriving first, second or simultaneously with a neighbour) of the respective species on **a** biomass, **b** number of flowerheads, and **c** final establishment (number of individuals established from ten sown seeds). Top panels **a**–**c** depict the five alien target species and the bottom panels the confamilial native target species as listed in Table 1. Bars are means ± SE

In the second part of the experiment, looking at priority effects, the order of arrival and species identity was relevant for all performance measures for both alien and native target species (Table 3). Alien species generally performed better when arriving first (mean ± SE: 9.29 ± 1.35 g of dry biomass; percent change compared to simultaneous arrival: + 45.6%) compared to arriving simultaneously (6.38 ± 0.74 g) with a neighbour species, while when arriving second (3.27 ± 0.66 g; − 48.7%) species performed worse than arriving simultaneously (Fig. 2). Native species suffered on average even more from late arrival (2.45 ± 0.60 g; − 64.7%) compared to simultaneous arrival (6.95 ± 0.75 g), whereas average differences to arriving first (6.00 ± 0.88 g; − 13.7%) are small. Similarly, for alien species the number of flowerheads was on average similar between arriving first (mean ± SE: 7.56 ± 1.49 number of flowerheads; percent change compared to simultaneous arrival: − 1.7%) and arriving simultaneously (7.69 ± 0.98) but smaller for arriving second (3.24 ± 0.86; − 57.9%). In contrast, for native species we find an interaction of order of arrival and species identity (Table 3). While *S. pumila* showed no difference between early and simultaneous arrival, compared to producing less flowerheads when arriving late compared to simultaneous (Fig. 2), *S. nigrum* benefits from arriving first compared to simultaneous, while we see no difference between late and simultaneous arrival. The other three native species (*C. album*, *C. diffusa* and *T. campestre*) had no apparent differences of order of arrival (note that for *T. campestre* no individuals of late arrival were recorded).

**Table 3:** Results of model selection of the priority experiment, showing the significant effects (*P* < 0.1) retained in the minimum adequate models

Performance measure	Status	Significant fixed terms	Test statistic	*R*^2^ marginal/adjusted	*R*^2^ conditional
Biomass	Alien	Order	χ^2^(*df* = 2, *N* = 109) = 30.1; *P* < 0.001	0.34	0.42
		Species	χ^2^(*df* = 4, *N* = 109) = 29.4; *P* < 0.001		
	Native	Order	χ ^2^(*df* = 2, *N* = 107) = 36.6; *P* < 0.001	0.51	0.56
		Species	χ ^2^(*df* = 4, *N* = 107) = 61.5; *P* < 0.001		
Flowerheads	Alien	Order	χ ^2^(*df* = 2, *N* = 109) = 49.7; *P* < 0.001	0.63	0.70
		Species	χ ^2^(*df* = 4, *N* = 109) = 102.1; *P* < 0.001		
	Native	Order*Species	χ ^2^(*df* = 7, *N* = 109) = 14.8; *P* = 0.040	0.57	0.60
Initial establishment	Alien	Order	*F*(2, 222) = 107.7; *P* < 0.001	0.61	
		Species	*F*(4, 218) = 35.3; *P* < 0.001		
	Native	Order	*F*(2, 222) = 64.4; *P* < 0.001	0.61	
		Species	*F*(4, 218) = 56.9; *P* < 0.001		
Final establishment	Alien	Order*Species	*F*(8, 216) = 2.5; *P* = 0.012	0.56	
	Native	Order*Species	*F*(8, 216) = 3.2; *P* = 0.002	0.62	
Flowering onset	Alien	Order*Species	χ ^2^(*df* = 6, *N* = 85) = 24.1; *P* < 0.001	0.81	0.81
	Native	Order*Species	χ ^2^(*df* = 7, *N* = 77) = 24.1; *P* < 0.001	0.34	0.43

For each model of the five performance measures (biomass, flowerheads, initial and final establishment, flowering onset), the significant fixed effects are shown with their respective test statistic and R^2^ of the models (marginal and conditional for biomass, flowerheads and flowering, adjusted R^2^ for initial and final establishment). Adjusted R^2^ for initial and final establishment was obtained using a Kullback–Leibler-divergence-based method, extended to quasi models by Zhang et al. (2017) using the R package rsq (Zhang 2021)

Furthermore, for final establishment and flowering onset the effect of order of arrival also depended on species identity, for both alien and native species (i.e., significant interaction, Table 3). For most species we see no apparent difference between first and simultaneous arrival in number of established individuals at harvest, but lower establishment of second arrival compared to simultaneous arrival. However, *A. retroflexus* additionally showed higher establishment for first arrival compared to simultaneous, while *C. diffusa* and *L. communis* showed no effect of order of arrival (Fig. 2). These trends were also identified for initial establishment (Electronic Supplementary Material, Fig. A3), while species differed much in the effect of order of arrival on flowering onset where no clear trend could be observed (Fig. A3). Interestingly, confamilial species pairs often performed similarly regardless of their status (Fig. 2, Fig A3). Although we could not statistically test this, visual inspection showed only minor differences between alien and native species (Fig. A4) in response to arriving early or late compared to simultaneous for most response variables. The main exception is for biomass production, whereby we find alien species (but not natives) to benefit from arriving first compared to simultaneous arrival.

## Discussion

### One alien neighbour vs two alien neighbour species

In the first part of our study, investigating the effects of number of alien neighbour species on a native plant, we found that native plants overall suffered more when having two alien neighbour species compared to one, producing relatively less biomass. A closer inspection of native target species showed a strong negative effect for the forbs *Chenopodium album* and *Lapsana communis* and a weak effect for *Solanum nigrum*. The strong negative effect multiple alien species can have on natives has been highlighted in a recent review (Kuebbing and Nuñez 2016) and involves a number of explanations. For instance, Flory and Bauer (2014) and Cushman et al. (2011) found experimental evidence for alien species facilitating the growth of other invasive species. Furthermore, across 190 alien species pairs, facilitation was observed in a quarter of cases (Ferenc and Sheppard 2020). Such facilitation between multiple alien species might then even lead to an accelerated negative effect on the native (‘invasional meltdown’, Simberloff and von Holle 1999). To date this hypothesis is not unequivocally supported.

Rather than accelerated effects of co-occurring alien species on natives, more commonly studies find additive effects of multiple alien species, such as Braga et al. (2020) find in aquatic ecosystems when comparing native communities with communities invaded by one, two or three alien species. Importantly, even if multiple aliens compete with rather than facilitate each other, effects of asymmetric competition may still lead to higher impacts on natives: if alien species have a higher negative effect on natives than on co-occurring aliens, this would lead to stronger negative effects on natives. This was also shown by a meta-analysis from Kuebbing and Nuñez (2016), who found alien species to have a nearly two times stronger negative effect on native species than on co-occurring other alien species. Another explanation why having two neighbour species compared to one is worse for the target might simply be due to a sampling effect, with two neighbours it is more likely that a good competitor is amongst the neighbours. There is also a higher chance of potentially greater overlap of niche space resulting in decreased performance. Which of the proposed mechanisms is at play is not possible to determine from our study design. However, we find more biomass per pot being produced by all neighbour plant combinations if two alien neighbour species are present compared to one and thus a more than additive negative effect of two species being present. Such increased neighbour biomass production may explain why some species experience strong negative effect of more than one neighbour species.

However, although the overall effect of the two neighbour treatment was significantly negative for biomass, we found strong species-specific effects for all response measures. The finding that particularly the native target species *C. album* and *L. communis* are strongly negatively affected (with strong negative log response ratios when growing with two alien neighbours) may be surprising given that these are highly successful species, abundant and widespread in Germany as well as particularly *C. album* being successful neophytes elsewhere (CABI 2021). In contrast, we find positive log response ratios for the native target species *Setaria pumila* (a grass) and *Trifolium campestre* (a legume), and for both a minor difference between treatments (one vs two alien neighbours). Generally, in line with such positive log response ratios, stronger intraspecific competition is more commonly expected compared to interspecific competition (Adler et al. 2018) when neglecting the often-assumed negative effect of alien competitors. In a similar experimental set up by Ferenc and Sheppard (2020) such stronger intraspecific competition (compared to interspecific competition by other aliens) was found for a few alien species (e.g., *Panicum capillare*, *Bidens pilosa*, *Cosmos bipinnatus*, *Vicia villosa)*, usually considered to be dominant or legume species. As *S. pumila* is a neophyte in North America and by being a grass species considered to be more competitive and dominant in resource uptake (Bloor et al. 2008), this might explain stronger intra- compared to interspecific competition. For *T. campestre*, log response ratios varied greatly and sample sizes were low, suggesting that these results should not be over-interpreted.

The factors that are related to these species-specific differences in the strength of the various native-alien and alien-alien interactions with their consequences should be further investigated in future studies. One potentially interesting avenue for further studies that our data hints at is the disappearance of potential positive effects of legume neighbours when growing with multiple aliens. Growing with only the legume *Vicia villosa* affected biomass and flowerheads of natives positively, whereas this effect diminished when a second alien neighbour was added (Electronic Supplementary Material, Figure A5). The positive effect in the one-neighbour treatment could be either simply because *V. villosa* is a weak competitor (although, in a previous study, we found it to be one of the dominant competitors, (Ferenc and Sheppard 2020)), or because as a nitrogen-fixing legume (Tate 1995) it may facilitate co-occurring species (Temperton et al. 2007). There are two possible explanations why this positive effect disappears when growing together with a second alien species. First, the second alien species might have had strong negative effects on the alien legume, so that the positive effect of nitrogen-fixation on the native plant was much reduced. In line with this explanation, we found that the alien legumes produced less biomass in the two-neighbour treatment than what would be expected from the single-neighbour treatment (Electronic Supplementary Material, Fig A6). The second possible explanation is similar to aforementioned asymmetric competition effects (Kuebbing and Nuñez 2016), whereby the second alien neighbour species benefits much more from the nitrogen-fixation, or from exploiting a weak competitor (accordingly, biomass of three of the four non-legume alien neighbour species was considerably higher in the two-neighbour treatment when growing with the legume than expected from the single-neighbour treatments, see Fig. A7). Given that we only had one legume in this study we cannot infer whether these are species-specific or functional group effects, however we suggest that this interesting pattern should be more thoroughly investigated in future studies shedding light on the role of (legume) facilitation in multi-species invasion scenarios.

### Arriving first, second or simultaneously

Concerning the second part of our study, how the order of arrival in the pot affects plant performance, we find a strong disadvantage of arriving second compared to simultaneously. Regardless of the short difference of only 3 weeks, the species arriving second produced less biomass and frequently less flowerheads. Also, fewer individuals germinated and were present at harvest when sown later than the neighbour. Differences between arriving first versus simultaneously with a neighbour were however less pronounced, and beneficial effects of early arrival mostly observed in alien species. Similarly Stuble and Souza (2016) showed a strong disadvantage of arriving later and only a small advantage of arriving early, while Dickson et al. (2012) and Goodale and Wilsey (2018) found strong advantages of arriving early especially for alien species. This suggests, along with Körner et al. (2008), who investigated priority effects of native species, altered arrival times can greatly affect species development and ultimately may alter community composition in subsequent years. Notably we used annual ruderal plant species in our experiment, in contrast to Dickson et al. (2012) or Goodale and Wilsey (2018) who used perennial species. Experiments commonly find annual ruderal species to be less competitive (Grime 1977) and hence such species might generally suffer more if a competitor is already established. Accordingly, we generally found a stronger disadvantage of arriving late (difference between late sown species and simultaneous treatment) compared to an advantage of arriving early (difference between early and simultaneously sown species), at least for natives.

One possible explanation for the commonly observed lack of advantage of arriving early might be favourable abiotic conditions in the experiment. Abiotic conditions such as nutrient availability (Kardol et al. 2013) or water depth in vernal pools (Collinge and Ray 2009) have been shown to pose a large effect on the magnitude of priority effects. However, we note that the greater magnitude of effects for arriving late compared to arriving first may also be due to the fact that species arriving later experienced a shorter experimental duration than early sown species as all species were harvested at the same time. Ideally, one might have accounted for this using a late sown simultaneous control, as some previous studies (Delory et al. 2019a) did. Nevertheless, we expect the short difference of 3 weeks between sowing events to have a small effect in our specific study system, as we used annual ruderal species which finish their life cycle within a short time span. While accounting for the difference in experimental duration would avoid confounding effects with the treatment of arrival time, with our approach we intended to mimic a more natural scenario: the end of the season will be the same for all individuals present in a certain site, regardless of differences in arrival time, similarly to the approach used by Dickson et al (2012). Additionally, our main interest was the response of species with different invasion status, whereby aliens and natives were equally influenced by the shorter experimental duration.

Although we could not directly statistically compare priority effects of aliens versus natives, responses of both status groups overall appear to be similar. However, in terms of biomass production, more alien compared to native species benefitted from arriving earlier compared to simultaneous (Fig. A4). This is in line with previous studies finding invasive species benefitted more from arriving earlier (Dickson et al. 2012; Goodale and Wilsey 2018). On the other hand, both alien and native species suffered from priority effects by an early arrived species, but the magnitude of this effect was on average even stronger for natives. Principally, differences might arise due to differing magnitudes of priority effects of the early arriving plant on the later arriving ones, or by a higher tolerance of such effects by the later arriving plant, as a previous study found (Stuble and Souza 2016). Notably, published studies comparing aliens and natives may be biased to some degree as studies often use common alien (invasive) species that are well known for their strong impacts (Hulme et al. 2013; but see Stuble and Souza 2016), but also mostly use rare natives to investigate effects by alien species (Vila and Weiner 2004). Zhang and van Kleunen (2019) compared the competitive ability of common and rare aliens versus common and rare natives and could not find an overall advantage of alien species. They showed that common natives would outcompete rare natives while common natives did not differ from common aliens, a result also in line with a previous study by Dawson et al. (2012). As we used common native species and their alien counterparts, this finding of commonness vs rarity being more relevant than alien vs native status might explain why we did not find great differences in priority effects between alien and native species in our study. Nevertheless, we find species-specific differences in magnitude of response to timing of arrival for some performance measures, and a tendency of related species to respond similarly to the treatment. While most species suffer from arriving second compared to simultaneous, only some species benefit from arriving early. The aliens *A. retroflexus*, *C. diffusa* and *P. capillare* as well as the native *S. nigrum* perform better when arriving first compared to simultaneously, which could hint at lower competitive ability for these species. However, even though *P. capillare* was greatly negatively affected by arriving second, it still produced the most biomass out of all species. In contrast, *C. diffusa* generally had a low rate of establishment, which may also explain why it has the lowest range size of all alien species used in this experiment.

## Conclusions

This study investigated two important yet not fully understood aspects of alien plant invasion. We found exacerbated negative effects of alien plants on at least some native species when growing together with two alien plant species compared to only one alien neighbour species, highlighting the importance of taking multiple invasions into account for management measures. The species-specific differences we found suggest interesting avenues for further research. Of particular importance could be the finding that the species showing strong responses were forbs that may suffer more from multiple alien species presence, while beneficial facilitative effects of certain neighbour species can disappear in the presence of multiple aliens. Although aliens more often benefitted from arriving first compared to simultaneous, we observed a general disadvantage of arriving second compared to simultaneous in a pot for both aliens and natives. Future studies might confirm whether these comparatively small differences between aliens and natives are indeed due to using common native species rather than rare species as often in such experiments. Our research provides insight into small-scale dynamics of invasion by co-occurring alien species from different families and different times of arrival. We observed relevant patterns affecting species interactions and proposed possible mechanisms behind them even in the comparably small set of species we investigated. Therefore, we expect future studies with a larger number of species to further enlighten the role of species-specific interactions or possible functional group effects such as by nitrogen-fixing legumes in sites invaded by multiple aliens and to achieve generalisations concerning the response of multiple alien and native plants to arrival time.

## Supplementary Information

Below is the link to the electronic supplementary material.

Supplementary file1 (PDF 1003 KB)

## Data Availability

Data are available from figshare. https://doi.org/10.6084/m9.figshare.15112365.

## Declarations

### Code availability statement

The R code is available from figshare. https://doi.org/10.6084/m9.figshare.15112365.
