# Supplementary material for: Native and alien species suffer from late arrival, while negative effects of multiple alien species on natives vary
Source: Oecologia. 2021 Aug 19;197(1):271–81. doi: 10.1007/s00442-021-05017-3 (PMC8445876; doi:10.1007/s00442-021-05017-3)
Supplement: Supplementary file 1 — Supplementary file1 (PDF 1003 KB) [file 442_2021_5017_MOESM1_ESM.pdf]

Native and alien species suffer from late arrival, while negative effects of multiple alien species on natives vary

Viktoria Ferenc*, Christian Merkert, Frederik Zilles, Christine S. Sheppard

Institute of Landscape and Plant Ecology, University of Hohenheim, 70593 Stuttgart

*Corresponding author: viktoria.ferenc@uni-hohenheim.de; Tel: +49 711 459 24086; Fax: +49 711 459 22831

Online Resource 1

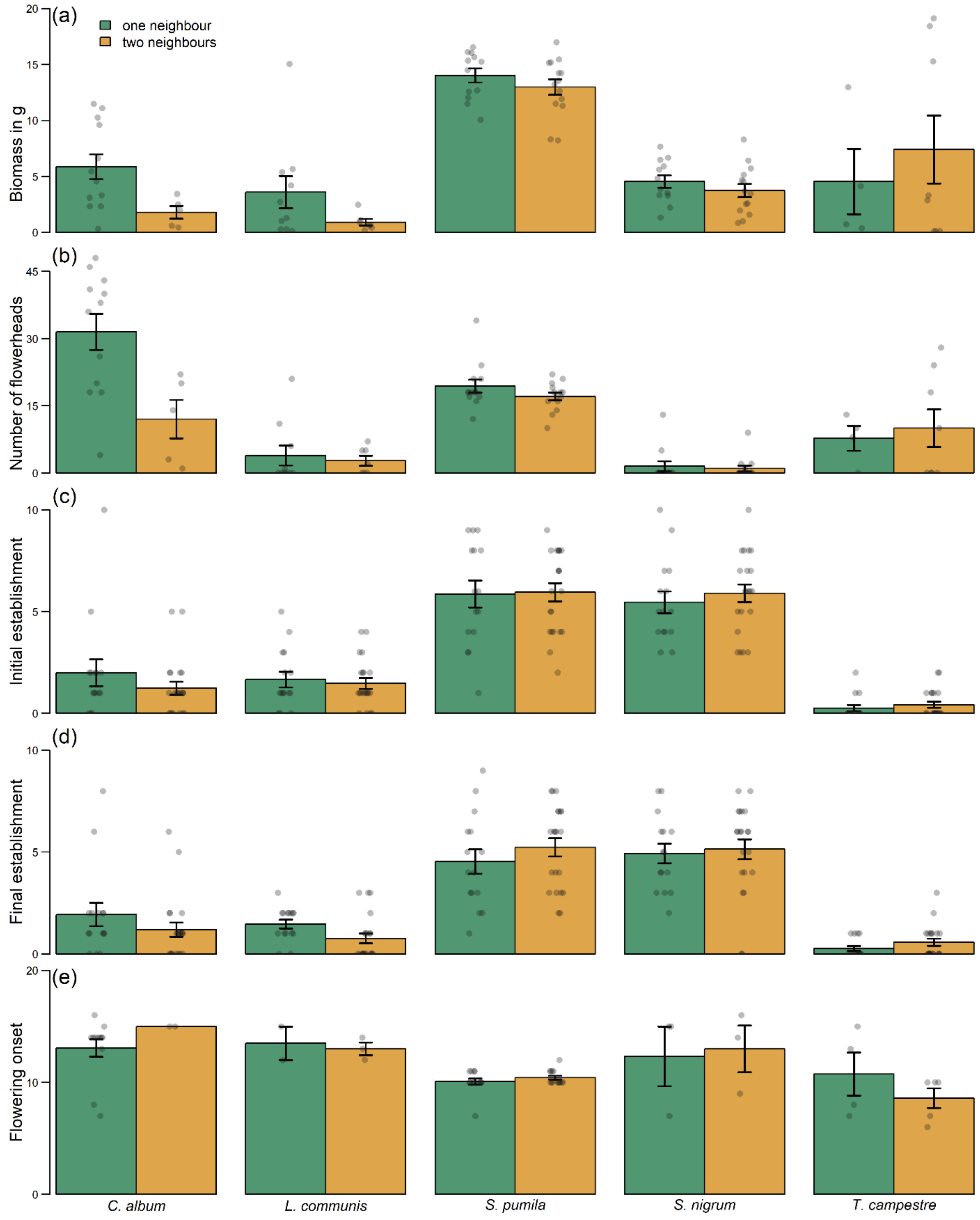

Fig. A1 Species-specific results of the neighbour-experiment. Barplots (means ± SE) show the effect of treatment (one vs two alien neighbour species) on the raw data for the performance measures a) biomass, b) number of flowerheads, c) initial and d) final establishment as well as e) flowering onset for the native target species *Chenopodium album*, *Lapsana communis*, *Setaria pumila*, *Solanum nigrum*, and *Trifolium campestre*. Grey dots depict individual datapoints. For sample sizes see Table A1.

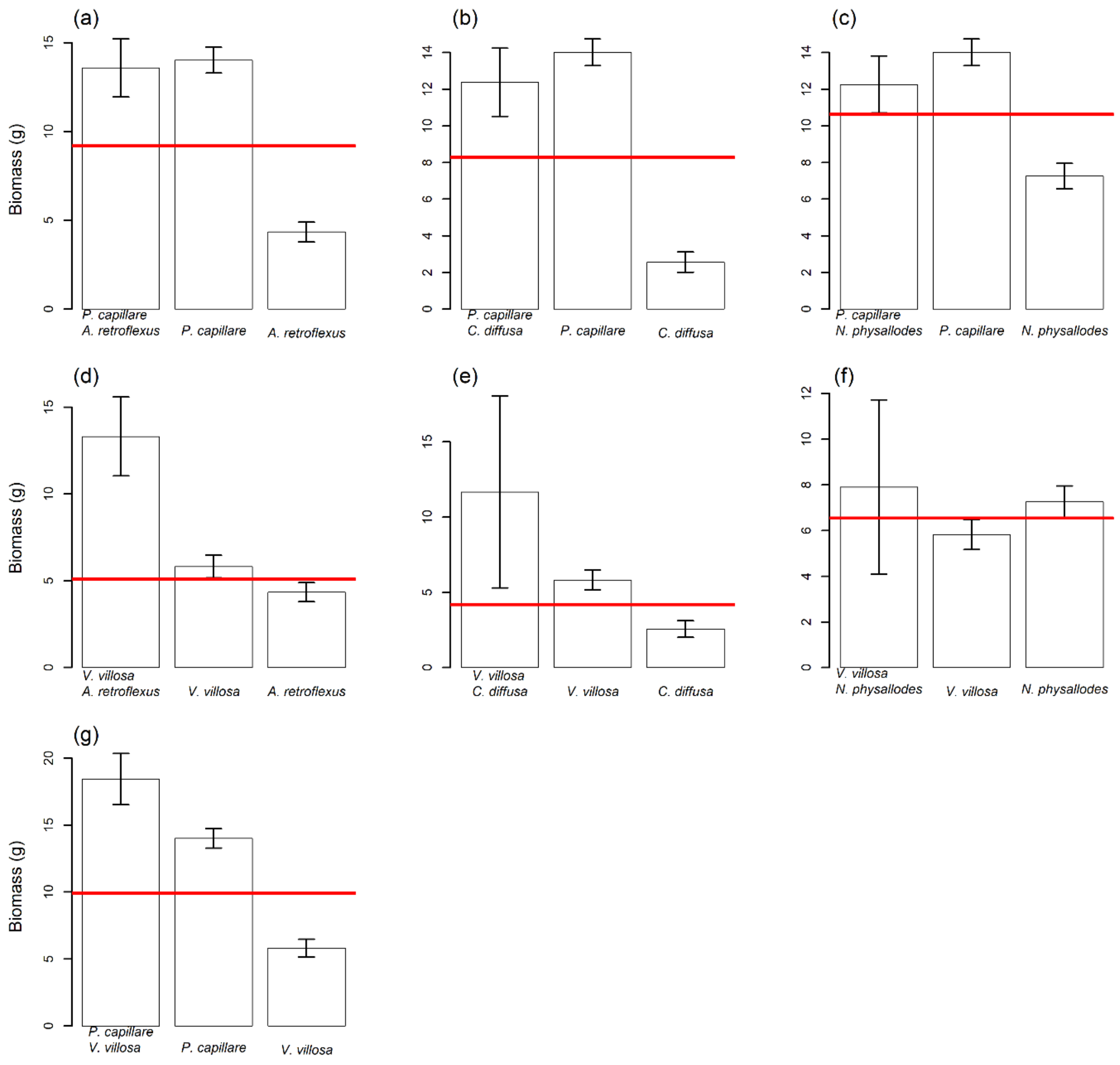

Fig. A2 Neighbour biomass in the neighbour-experiment. Barplots (means ± SE) show the biomass production (g) of alien neighbours. Each panel a) – g) shows data of a specific alien neighbour species combination (first bar) and the two corresponding single neighbour species treatments (second and third bar). The red line indicates the expected biomass production from the average of the single neighbour species treatments. The actual biomass reached in the two-neighbour treatment (first bar) always exceeds the expected biomass. Note that in all neighbour treatments, a total of ten alien seeds were sown.

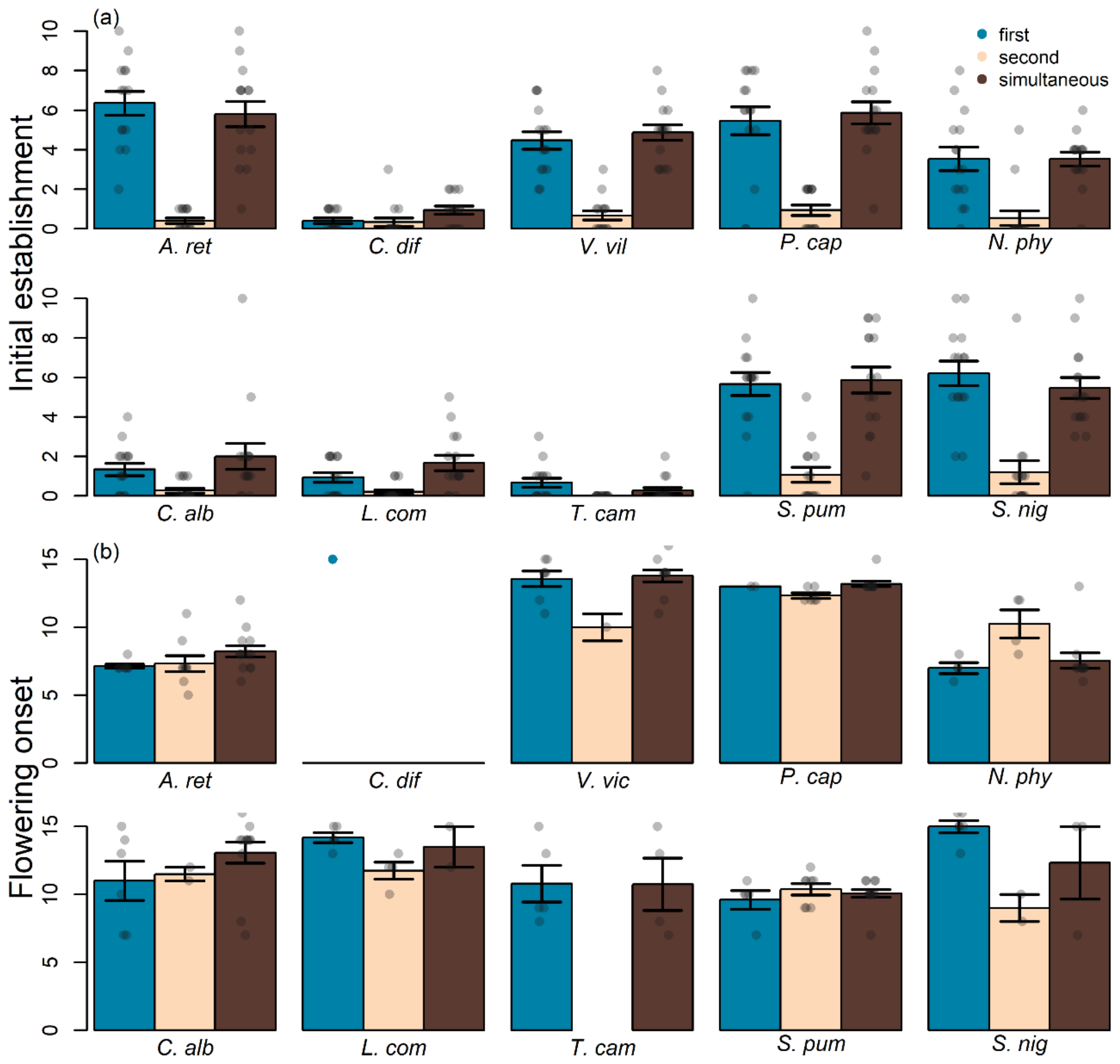

Fig. A3 Results of the priority-experiment. Barplots (means ± SE) show the effect of order of arrival (arriving in the pot first, second or simultaneously with a neighbour) of the respective species on the performance measures a) initial establishment (number of individuals established three weeks after sowing from 10 sown seeds) and b) flowering onset. Top panels for a) and b) depict the five alien target species and the bottom panels the confamilial native target species as listed in Table 1. Dots depict individual datapoints. For sample sizes see Table A2.

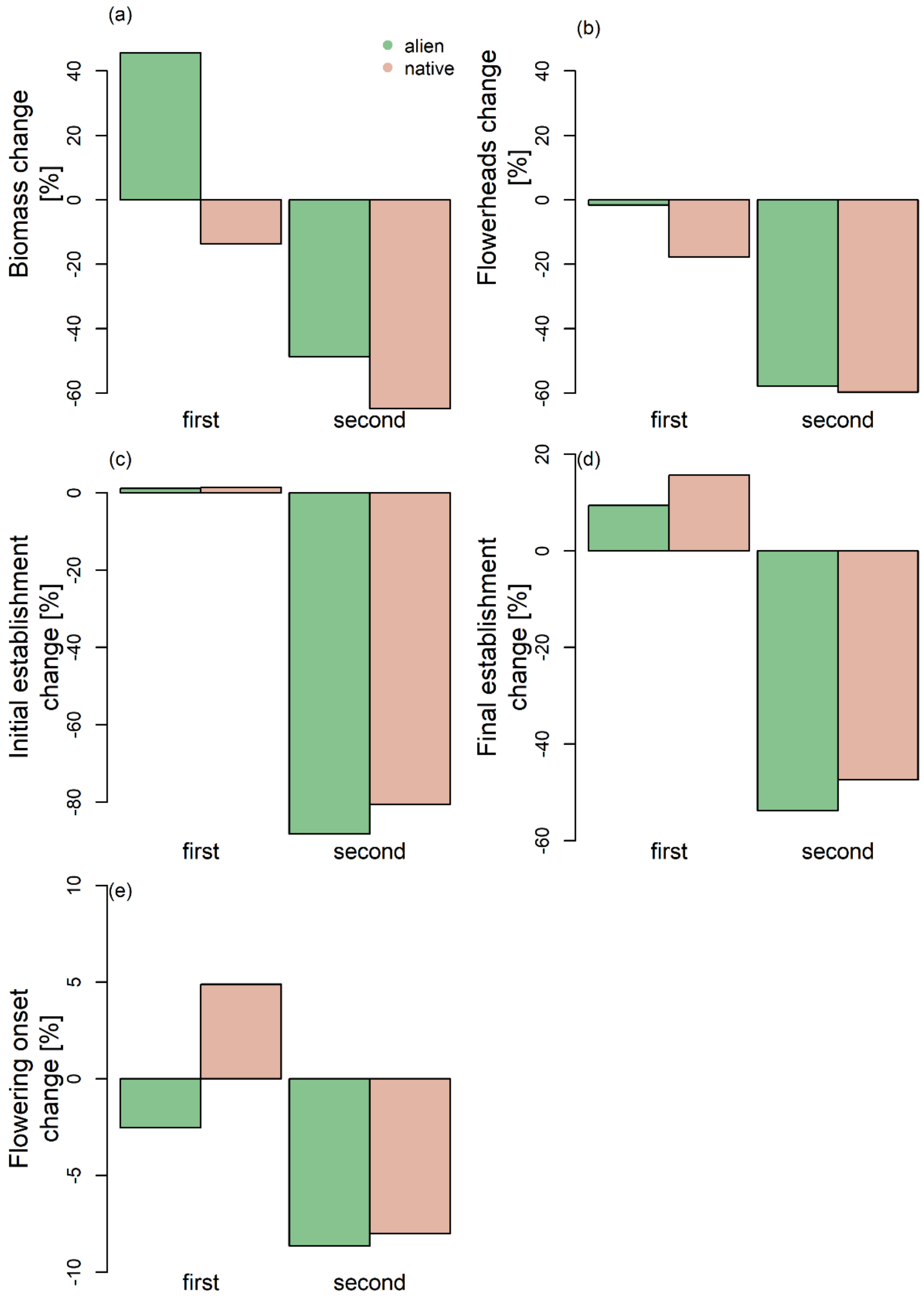

Fig. A4 Results of the priority experiment: comparison of the magnitude of effects on alien versus natives. Barplots show the average percentage change of arriving first (second, respectively) relative to simultaneous arrival for the five performance measures a) biomass, b) number of flowerheads, c) initial establishment, d) final establishment and e) flowering onset.

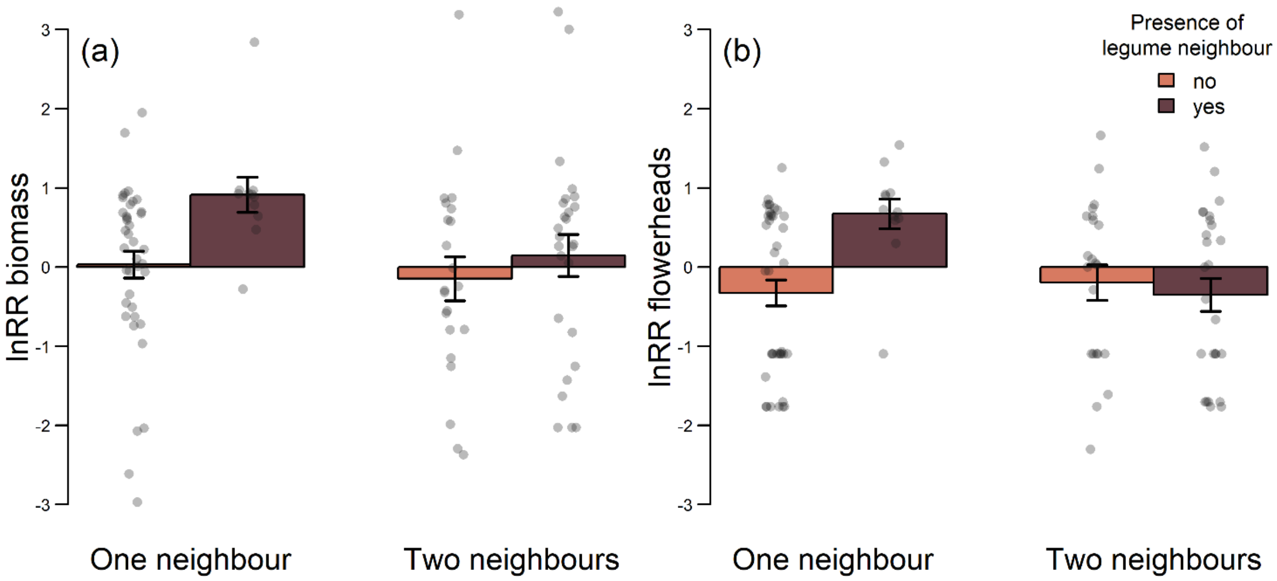

Fig. A5 Results of the neighbour-experiment with regard to presence of a legume neighbour. Barplots (means ± SE) show the effect of treatment (one vs. two alien neighbour species) and presence of the legume neighbour *Vicia villosa* on the performance measures a) lnRR biomass and b) lnRR flowerheads. Grey dots depict individual data points, with sample sizes 39, 11, 22, 26 (a, from left to right) and 39, 12, 22, 26 (b).

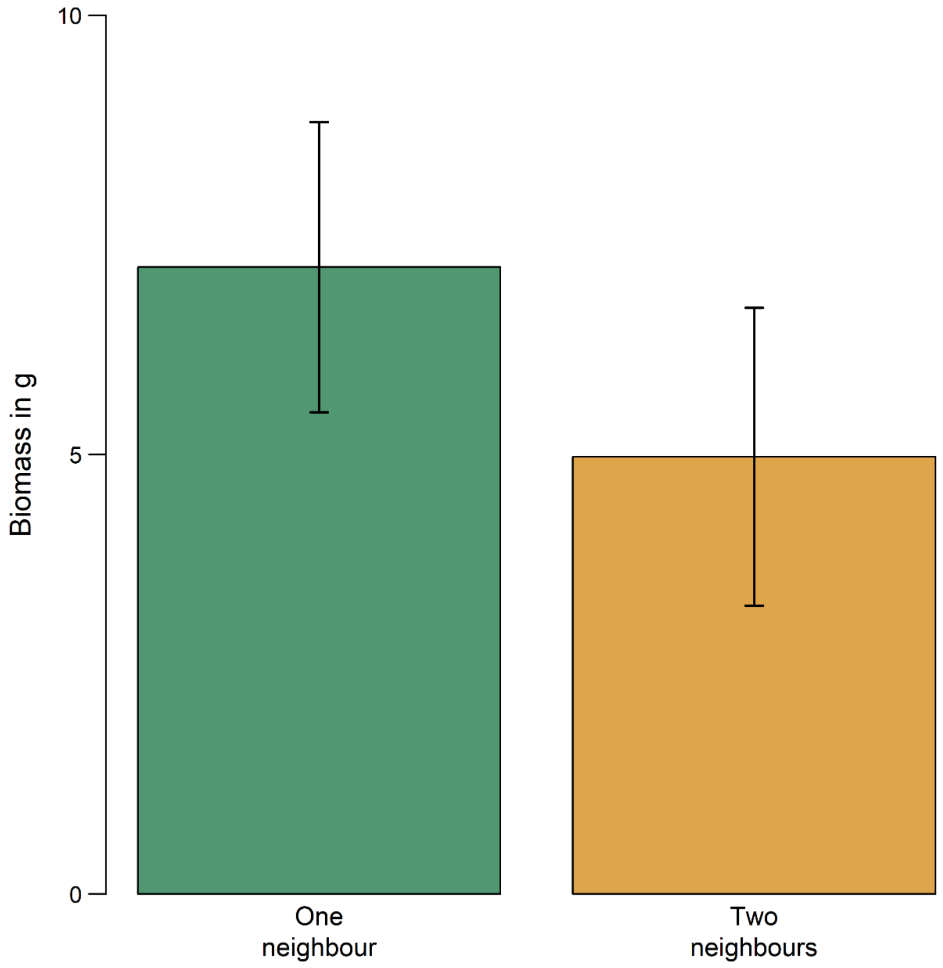

Fig. A6 Results of the neighbour experiment: possible mechanisms for the disappearance of the beneficial legume effect with two alien neighbour species. Barplots (means ± SE) depict alien legume biomass (*V. villosa*) either alone with the native (biomass divided by two due to double amount of seeds of the alien species, green bar) or with a second alien neighbour (orange bar), averaged across all neighbours and natives.

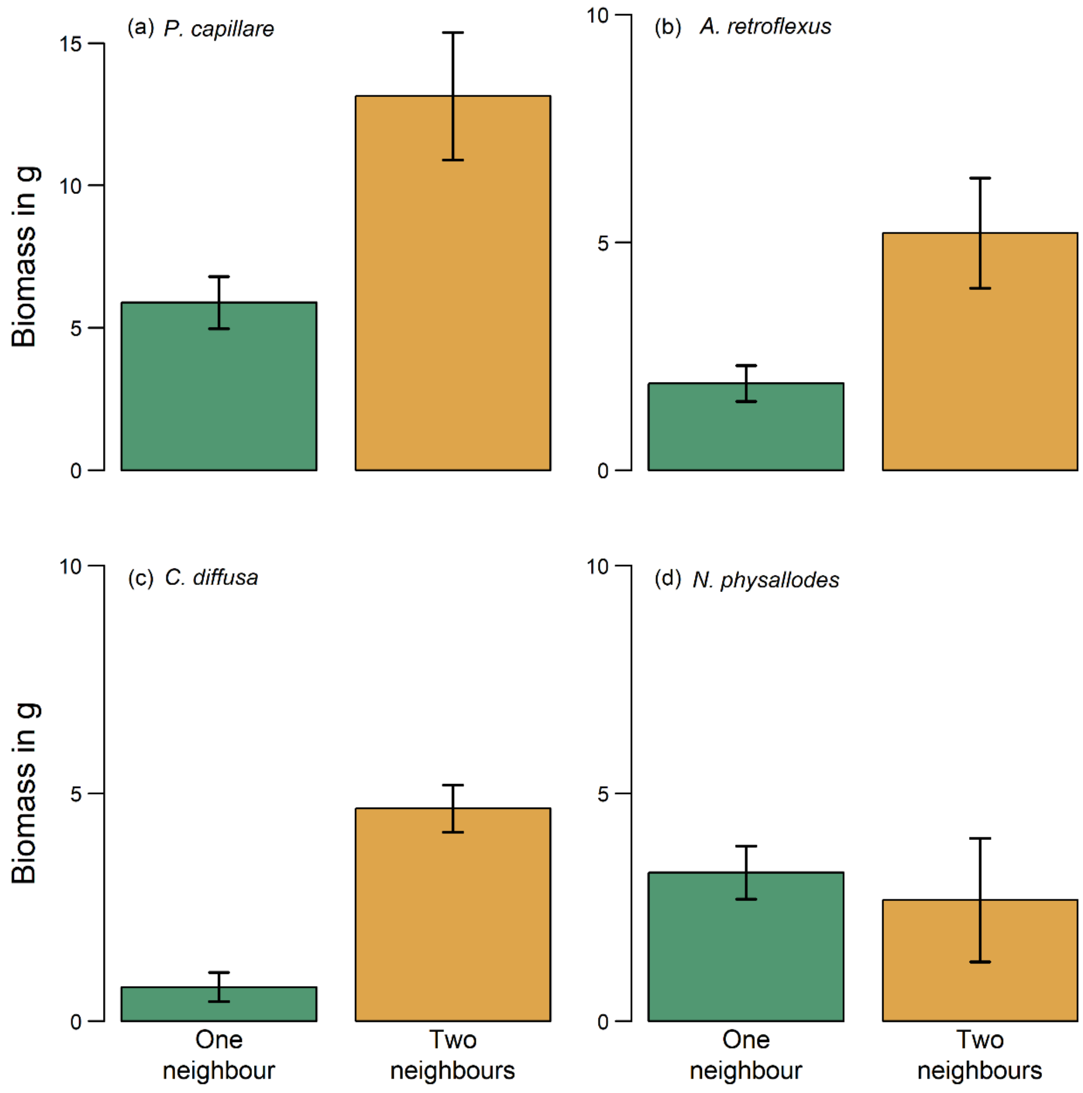

Fig. A7 Results of the neighbour experiment: possible mechanisms for the disappearance of the beneficial legume effect with two alien neighbour species. Barplots (means ± SE) depict biomass of the alien neighbour (a-d), averaged across all native target species, when growing with only the native (biomass divided by two due to double amount of seeds of the alien species, green bars), or with an additional alien neighbour, specifically with the legume *V. villosa* (orange bars).

Table A1 Number of observations used for analysis of the neighbour-experiment part for each species for the five performance measures

Species	Number of neighbour species	Biomass	Number of flowerheads	Initial establishment	Final establishment	Flowering onset
Chenopodium album	One neighbour	12	12	15	15	12
	Two neighbours	5	5	21	21	2
Lapsana communis	One neighbour	10	10	15	15	2
	Two neighbours	7	7	21	21	3
Setaria pumila	One neighbour	12	13	15	15	13
	Two neighbours	14	14	21	21	14
Solanum nigrum	One neighbour	12	12	15	15	3
	Two neighbours	14	14	21	21	3
Trifolium campestre	One neighbour	4	4	15	15	4
	Two neighbours	8	8	21	21	5

Table A2 Number of observations used for analysis of the priority effects part for each species in each order of arrival treatment for the five performance measures

Species	Order of arrival	Biomass	Number of flowerheads	Initial establishment	Final establishment	Flowering onset
Amaranthus retroflexus	first	7	7	14	14	7
	second	11	11	15	15	9
	simultaneous	13	13	15	15	13
Centaurea diffusa	first	4	4	15	15	1
	second	3	3	15	15	0
	simultaneous	5	5	15	15	0
Nicandra physalodes	first	4	4	15	15	4
	second	4	4	15	15	4
	simultaneous	11	11	15	15	11
Panicum capillare	first	2	2	15	15	2
	second	6	6	15	15	6
	simultaneous	10	10	15	15	10
Vicia villosa	first	8	8	15	15	7
	second	9	9	15	15	1
	simultaneous	12	12	15	15	10
Chenopodium album	first	6	6	15	15	6
	second	2	2	15	15	2
	simultaneous	12	12	15	15	12
Lapsana communis	first	7	7	15	15	5
	second	9	9	15	15	4
	simultaneous	10	10	15	15	2
Setaria pumila	first	5	5	15	15	5
	second	8	8	15	15	8
	simultaneous	12	13	15	15	13
Solanum nigrum	first	9	9	15	15	6
	second	5	6	15	15	2
	simultaneous	12	12	15	15	3
Trifolium campestre	first	6	6	15	15	5
	second	0	0	14	14	0
	simultaneous	4	4	15	15	4
